# Ileocaecal Intussusception Due to Burkitt Lymphoma: A Surgical and Haematological Emergency

**DOI:** 10.7759/cureus.50904

**Published:** 2023-12-21

**Authors:** Mostafa Elkhawaga, Bryony Ross, Ella Sugo, Chelsea Reedman-Hawes, Hasitha Balasuriya

**Affiliations:** 1 Surgery, John Hunter Hospital, Newcastle, AUS; 2 The Children's Cancer and Haematology Service, John Hunter Children's Hospital, Newcastle, AUS; 3 Anatomical Pathology, John Hunter Hospital, Newcastle, AUS; 4 Department of Colorectal Surgery, John Hunter Hospital, Newcastle, AUS

**Keywords:** intussusception, lymphoma, abdominal pain differential diagnosis, burkitt, intestinal obtstruction

## Abstract

Intussusception is the protrusion and invagination of a segment of the bowel, referred to as the intussusceptum, into another adjacent segment known as the intussuscipiens. The age range during which intussusception is most observed in children is between three and 18 months. Unlike intussusceptions in children, where the cause is often idiopathic, a specific underlying factor is discernible in a significant majority of cases among adults. In this article, we will present a case of intussusception in an adolescent caused by Burkitt lymphoma.

## Introduction

Intussusception was initially documented in 1674 by Barbette of Amsterdam [1]. Intestinal intussusception is notably more prevalent among paediatric patients than adults, with a ratio of 20:1 [2]. Intussusception in adults is regarded as an infrequent condition, representing merely 5% of all instances of intussusceptions and approximately 1-5% of cases of bowel obstruction [3]. The most frequently reported presenting symptom of intussusception is abdominal pain, followed by vomiting and rectal bleeding [4]. Adult patients often experiencing intestinal intussusception report non-specific abdominal pain as their primary complaint. To establish an accurate diagnosis, a high level of clinical suspicion is required, typically validated through the use of abdominal computerised tomography (CT) imaging [5].

## Case presentation

Our patient is a 17-year-old male who was referred to the emergency department with an outpatient ultrasound suggesting intussusception. He reported a two-week history of intermittent, colicky right-sided abdominal pain, vomiting, obstipation, and unintentional weight loss. He had no haematochezia. His medical background includes a traumatic brain injury with subsequent mild intellectual impairment, and his family history was unremarkable. On examination, the patient was hemodynamically normal, stable, and was afebrile. His abdominal examination demonstrated mild tenderness in the right iliac fossa with no peritonism.

Laboratory tests are detailed in Table 1. The results showed an elevated C-reactive protein of 48 mg/L and were otherwise unremarkable, including renal function, carcinoembryonic antigen (CEA), and, notably, given the subsequent diagnosis, lactate dehydrogenase.

**Table 1:** Laboratory investigations at the time of presentation. CEA, carcinoembryonic antigen

Laboratory investigations	Results	Normal range
CEA	<1 ug/L	<5 ug/L
White cell count	7.9 x 10^9^/L	4-11 x 10^9^/L
Neutrophils	4.7 x 10^9^/L	2-8 x 10^9^/L
Haemoglobin	164 g/L	130-180 g/L
C-reactive protein	48 mg/L	<5 mg/L
Bilirubin	10 umol/L	<20 umol/L
Creatinine	65 umol/L	60-110 umol/L
Lactate dehydrogenase	178 U/L	130-250 U/L

**Figure 1:**
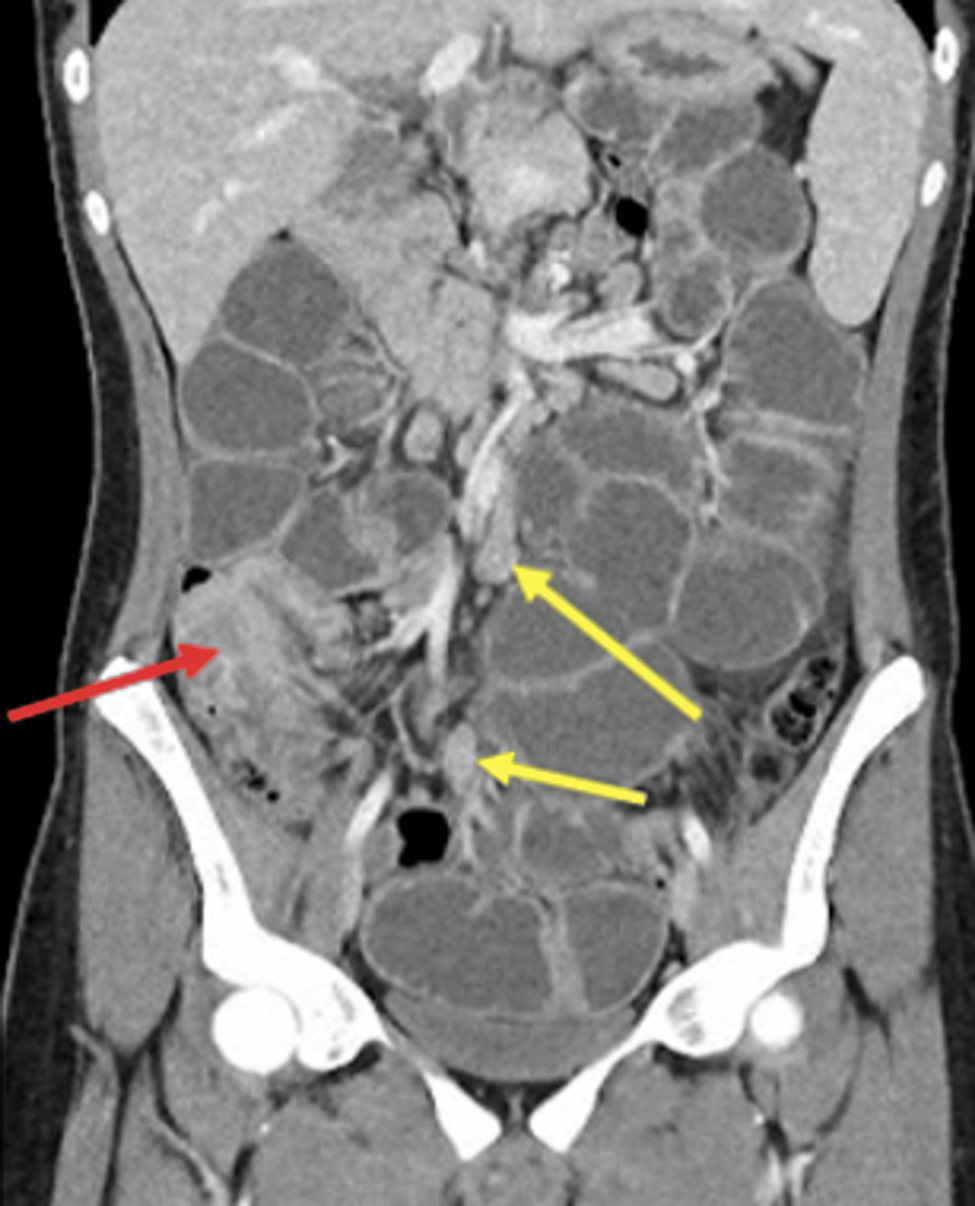
Ileocaecal intussusception (red arrow) with lymphadenopathy (yellow arrows).

His CT scan showed a small bowel obstruction secondary to ileocaecal intussusception and multiple enlarged lymph nodes seen throughout the central abdomen (Figure 1).

**Figure 2:**
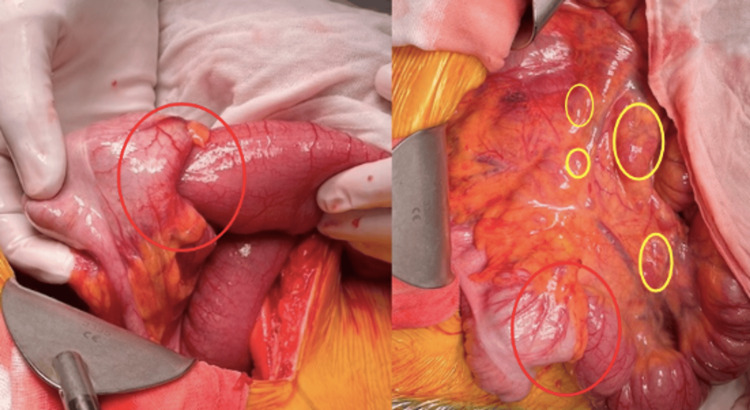
Intraoperative findings: Ileocaecal intussusception (red circles) and mesenteric lymph node involvement (yellow circles).

**Figure 3:**
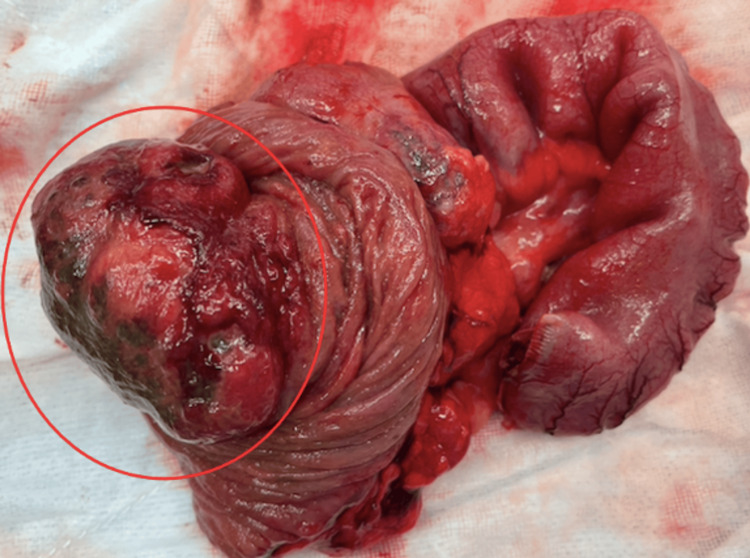
Terminal ileum intussuscepted tumour mass (red circle).

The patient proceeded to a laparotomy and right hemicolectomy with primary anastomosis. High ligation of the ileocolic pedicle was performed, taking care not to reduce the intussusception. Intraoperative findings revealed an ileocaecal intussusception with extensive mesenteric lymphadenopathy (Figure 2). The lumen was inspected following resection (Figure 3).

**Figure 4:**
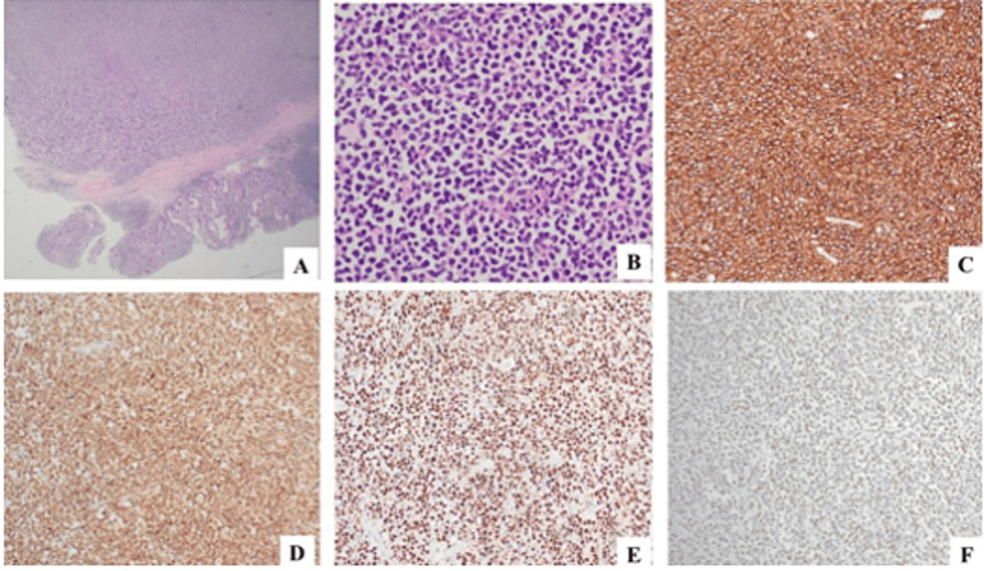
(A) Low-power image of lesion with overlying mucosa (H&E 200×). (B) High-power image of lesion demonstrating moderately pleomorphic vesicular nuclei with a small amount of eccentric basophilic cytoplasm (H&E 400×). (C) CD20 positive strong staining (200×). (D) CD10 positive moderate staining (200×). (E) Ki67 approaching 100% positive tumour nuclei (200×). (F) C-MYC positive widespread moderate staining (200×).

Histopathological examination demonstrated a 55 mm, ulcerated, transmural tumour confined to the intussusception lead point mass within the terminal ileum. The tumour was seen to infiltrate mesenteric fat; however, there was no lymph node involvement, supported by negative immunohistochemistry staining. Architectural and cytological features of the tumour included sheets of medium-sized, atypical lymphoid cells with moderately pleomorphic vesicular nuclei, and often multiple, small basophilic nucleoli. The tumour had a high proliferation index with numerous mitoses, abundant apoptotic debris, and a Ki67 (mitotic index) approaching 100% of tumour nuclei. An immunohistochemical panel revealed positive pan B lymphocyte markers CD20, CD79a, and PAX5; negative pan T cell markers CD3 and CD5; a negative TdT; positive CD10; and widespread moderate intensity C-MYC staining in majority of tumour cells. The morphological features and immunohistochemical profile were consistent with Burkitt lymphoma (Figure 4). The tissue was then forwarded to SydPath for molecular confirmation by FISH, which identified an IgH-MYC rearrangement compatible with the histological diagnosis of Burkitt lymphoma.

Upon receiving the histological diagnosis, an urgent haematology opinion was sought. Postoperative positron emission tomography (PET) scan showed reactive changes post-surgery, bulky spleen, and bone marrow uptake likely reactive, and an intensely metabolically active mesenteric node at the axial level of L3/L4 suspicious of persistent metabolically active lymphoma. His cerebrospinal fluid (CSF) did not contain malignant cells. Inpatient treatment with high-dose steroids and chemotherapy was commenced as per the Children’s Oncology Group protocol ANHL1131 (R-COPADM) (rituximab, cyclophosphamide, Oncovin (vincristine), prednisone, Adriamycin (doxorubicin), and methotrexate) [6] on postoperative day six.

The patient developed a superficial wound infection, managed with negative pressure wound therapy, and was subsequently discharged. He was represented on the 17th postoperative day (following induction chemotherapy) with a large collection in the right iliac fossa. Percutaneous drainage with culture-guided antibiotic cover was performed, and serial imaging demonstrated complete resolution with non-operative management. Chemotherapy was briefly ceased (for 14 days), and when the patient was clinically stable, he was treated with three cycles of R-Gem-Ox (rituximab, gemcitabine, and oxaliplatin) [7], following a recent publication by Bender et al. demonstrating effectiveness in children and adolescents with NHL who were unfit for intensive therapy. On the complete resolution of the dehiscence and wound infection, he was subsequently able to complete ANHL1131 without incident.

Both interim and post-completion of therapy PET were negative, and abdominal ultrasound three months post-therapy was also reassuring. Our patient remains on regular follow-up and has gone back to school, work, and all his activities of daily living.

## Discussion

Intestinal intussusception may be limited to the small intestine (entero-enteric), limited to the large bowel (colo-colic), or characterised by prolapse of the terminal ileum into the caecum (ileo-caecal) [8]. It may be idiopathic or secondary to benign or malignant pathology [9]. Approximately 1.5-12.0% of intussusception cases in individuals can be attributed to identifiable underlying pathological causes [10]. Benign lesions are the more commonly encountered type of lesions in the small intestine, whereas malignant lesions make up to 30% of all cases of intussusception in this region [11]. Lymphoma is a relatively rare cause of intussusception in children, accounting for only 6.5% of cases. Non-malignant intussusception is more common in paediatric patients with the classic triad of symptoms, including cramping abdominal pain, bloody diarrhoea, and the presence of a tender, palpable mass. The clinical presentation of adult intussusception varies, with non-specific symptoms such as nausea, vomiting, gastrointestinal bleeding, constipation, and abdominal distension [12]. The presence of prolonged symptoms and unexplained weight loss can be indicative of gastrointestinal lymphoma [13], as noted in the presented case. B-cell malignant lymphomas are the most common type of lymphoma found in the small intestine, with Burkitt lymphoma accounting for approximately 9% of all intestinal lymphomas [14]. Burkitt lymphoma typically presents with widespread disease and may present with life-threatening tumour lysis syndrome with hyperkalaemia, renal failure, and elevated lactate dehydrogenase (none of which were thankfully seen in our patient). It has a tumour doubling rate of 25 hours and requires prompt diagnosis and treatment [15]. The lack of widespread disease, the absence of tumour lysis in our patient, and the unusual age at presentation add to the atypical presentation in this case.

## Conclusions

This case of Burkitt lymphoma, causing ileocaecal intussusception, is a rare surgical and haematological emergency. The case highlights the importance of urgent surgery, clinical suspicion, pathological evaluation, and prompt liaison with haematology to commence treatment to reduce morbidity and mortality. It also highlights that these patients can be susceptible to further surgical complications due to the nature of their underlying disease and the multidisciplinary care that they often continue to require to complete therapy successfully.
